# DIAPHRAGMATIC STRENGTHENING EXERCISES FOR PATIENTS WITH POST COVID-19 CONDITION AFTER MILD-TO-MODERATE ACUTE COVID-19 INFECTION: A RANDOMIZED CONTROLLED STUDY

**DOI:** 10.2340/jrm.v56.25491

**Published:** 2024-06-11

**Authors:** Tamer I. ABO ELYAZED, Ahmed ABD EL-MONEIM ABD EL-HAKIM, Ola I. SALEH, Marwa Mostafa Fadel SONBOL, Hoda Assad EID, Eman M. MOAZEN, Mohammad Hamad ALHASSOON, Seham Ezzat Fathy ELFEKY

**Affiliations:** 1 Physical Therapy For Internal Medicine Department, Faculty of Physical Therapy, Beni-Suef University, Ben-Suef, Egypt; 2 Basic Sciences Department, Faculty of Physical Therapy, Beni-Suef University, Ben-Suef, Egypt; 3 Diagnostic Radiology Department, Al-Azhar University, Cairo, Egypt; 4 Chest Diseases Department, Al-Azhar University, Cairo, Egypt; 5 Internal Medicine Department, King Fahd Specialist Hospital, Burydah, Saudi Arabia; 6 Chest Diseases Department, Tanta University, Tanta, Egypt

**Keywords:** post COVID-19 condition, incentive spirometry, deep breathing

## Abstract

**Objective:**

To assess the clinical effects of incentive spirometry (IS) and diaphragmatic breathing (DB) in patients with post COVID-19 condition and diaphragmatic dysfunction as compared with the standard care alone.

**Methods:**

The present longitudinal randomized study included 60 patients with post COVID-19 condition and diaphragmatic dysfunction. Patients were equally randomized to receive standard care plus IS (G1), standard care plus DB (G2) or standard care alone (G3) for 8 weeks. The primary outcome is clinical improvement as evaluated by the modified Medical Research Council (mMRC) dyspnoea scale.

**Results:**

Comparison between the studied groups revealed significant improvement in G1 and G2 in all parameters at the end of follow-up. However, no significant improvement was found in G3. At the end of follow-up, 15 patients (75.0%) in G1, 11 patients (55.0%) in G2, and 3 patients (15.0%) in G3 showed improvement on the mMRC dyspnoea scale. Multivariate logistic regression analysis identified mild acute COVID-19 infection (*p* = 0.009), use of IS (*p* < 0.001), and use of DB (*p* = 0.023) as significant predictors of improvement on the mMRC dyspnoea scale.

**Conclusions:**

IS or DB training in addition to the standard care in post COVID-19 condition was associated with better clinical improvement as compared with the standard care alone.

LAY ABSTRACT

The present study assessed the value of respiratory muscle exercises in the form of incentive spirometry and deep breathing in patients with post COVID-19 condition. The study concluded that these exercises are useful for patients with shortness of breath after acute COVID-19 infection.

Post COVID-19 condition refers to persistence of 1 or more COVID-19 symptoms 3 months after seroconversion of acute COVID-19 infection (1). While the incidence of post COVID-19 condition is usually higher in patients with severe acute disease, even mild infection can be followed by long-term sequalae (2). Symptoms of post COVID-19 condition may include cough, dyspnoea, fatigue, palpitations, insomnia, chest pain, decreased or lost appetite, abdominal discomfort, arthralgia with or without myalgia, paraesthesia, hair loss, hearing loss, or tinnitus (3). The potential mechanisms for this phenomenon include viral persistence, chronic low-grade inflammatory response, autoimmunity, immune dysregulation, persistent endothelial dysfunction, coagulopathy, and molecular mimicry (4, 5).

Diaphragmatic dysfunction is commonly seen in patients with post COVID-19 condition (6). They frequently have impaired resting and exertional breathing pattern, and are more prone to diaphragmatic fatigue even in the presence of normal spirometry (7). Current evidence recommends exercise training or respiratory muscle training for post COVID-19 condition (8). Respiratory muscle training interventions have proved to be effective in management of many conditions with pulmonary affection. These include obstructive sleep apnoea (9), neuromuscular disease (10), asthma (11), stroke (12), chronic obstructive pulmonary disease (13), and spinal cord injury (14). Incentive spirometry (IS) is a commonly used tool for respiratory muscle training in miscellaneous conditions including COVID-19 (15–17). Diaphragmatic breathing (DB) exercises include a variety of protocols that have been efficiently used in patients with chronic obstructive pulmonary disease (18), gastroesophageal reflux disease (19), and COVID-19 (20).

The present randomized controlled study aimed to assess the clinical effects of IS and DB in patients with post COVID-19 condition with diaphragmatic dysfunction.

## METHODS

The present longitudinal randomized study was conducted at Al-Azhar University Hospitals. The study protocol was approved by the ethical committee of Al-Azhar Faculty of Medicine and all patients provided informed consent before participation. All patients were instructed on the purpose of the study, alternative therapies, possible risks, and expected outcome. The study included 60 patients with post COVID-19 condition and diaphragmatic dysfunction recruited from the outpatient clinic of the chest diseases department. All patients had seroconversion from confirmed mild-to-moderate COVID-19 infection and presented with easy fatiguability and/or shortness of breath and/or cough. Patients were excluded if they had chronic lung disease, neuromuscular disorder, diaphragmatic hernia, malignancy, phrenic nerve injury, abdominal or thoracic surgery, traumatic diaphragmatic injury, severe malnutrition, or obesity.

At baseline, all patients were subjected to careful history taking, thorough clinical examination, pulsed oximetry, and standard laboratory assessment. Evaluated pulmonary functions included forced expiratory volume in the 1st second (FEV1), slow vital capacity (SVC), forced vital capacity (FVC), and maximal voluntary ventilation (MMV). The physical performance of the patients studied was assessed using a 6-minute walk test (6MWT).

The diaphragm was assessed during tidal, deep, and sniff breathing using an ultrasound scanner equipped with 3.5 MHz curvilinear and 8 MHz linear probes (SSI6000, Sonoscape, Nanshan, China) for diaphragmatic excursion (M-mode) and thickness (B-mode). Only the right diaphragm was assessed. It was found to be sufficient and more precise for diagnosis of diaphragmatic dysfunction in comparison with the left side for technical considerations (21). All sonographic measurements were made at baseline and at the end of follow-up by the same sonographer. Patients were diagnosed with diaphragmatic dysfunction if they had diaphragmatic excursion of < 10 mm or paradoxical diaphragmatic movement and/or if they had diaphragm thickening fractions < 20% (22–24). In addition, performance of daily activities was assessed using the modified Medical Research Council (mMRC) dyspnoea scale. This is a self-assessment tool that measures the impairment caused by breathlessness during daily activities. mMRC is rated on a scale from 0 to 4 (25).

Patients were equally randomized to receive standard care plus IS (G1), standard care plus DB (G2), or standard care alone (G3) using computer-generated random tables and sealed envelope technique. While patients and investigators were not blinded regarding patients’ allocation to interventional groups, all assessments were performed by independent nurses or technicians who were not aware of the study purpose or patients’ allocation.

Standard care included avoidance of smoking and alcohol and exposure to dust or respiratory irritants, a balanced diet, and daily regular exercise. A physiotherapist suggested multiple forms of exercises for patients according to their clinical condition, current physical fitness and exercise habits before COVID. The most important concern was the post-exertional exaggeration of symptoms. Patients were advised to be cautious and were thoroughly monitored throughout the study. In addition, they were educated regarding the warning signs that may indicate critical worsening of the condition and were encouraged to have COVID-19 vaccination if not received. IS training was performed at home using the UNA01 flow-oriented incentive spirometer (UNICARE, China) after proper education of patients at the hospital. The IS training protocol consisted of use of incentive spirometers for 10–15 min twice daily for 8 weeks (26). DB exercises included self-positioning in the prone position on the elbows and diaphragmatic breathing for 10–15 min twice daily for 8 weeks.

During the 8-week follow-up period, all patients were observed daily. They showed excellent compliance with the study interventions. Finally, all patients were reassessed. The primary outcome is clinical improvement as evaluated by the mMRC dyspnoea scale. Data obtained from the present study were presented as number and percent or mean and standard deviation (SD) and compared using a chi-square test or one-way ANOVA with post-hoc Tukey’s test analysis. Binary logistic regression analysis was used to identify predictors of improved mMRC dyspnoea scale. All statistical computations were processed using SPSS version 27 (IBM Corp, Armonk, NY, USA) with *p*-value < 0.05 considered statistically significant.

## RESULTS

The present study included 60 patients with post COVID-19 condition and diaphragmatic dysfunction. Patients were randomly and equally allocated to the 3 treatment groups. All groups are comparable regarding the baseline characteristics including age, sex distribution, body mass index (BMI), COVID-19 severity, time since covid-19 seroconversion, and radiological findings (Table I).

**Table I:** Baseline characteristics in the groups studied

Factor	G1 Standard care + IS *n* = 20	G2 Standard care + DB *n* = 20	G3 Standard care *n* = 20	*p*-value
Age (years), mean±SD	39.8 ± 4.7	38.7 ± 4.2	40.4 ± 5.4	0.51
Male/female, *n*	12/8	12/8	10/10	0.76
BMI (kg/m^2^), mean±SD	24.3 ± 2.3	23.1 ± 3.1	24.0 ± 1.8	0.31
COVID-19 severity, *n* (%)				
Mild	12 (60.0)	15 (75.0)	11 (55.0)	0.39
Moderate	8 (40.0)	5 (25.0)	9 (45.0)	
Time since COVID-19 seroconversion (months), mean±SD	3.9 ± 0.8	4.0 ± 0.8	3.8 ± 0.8	0.72
Radiological findings, *n* (%)				
Free	11 (55.0)	13 (65.0)	10 (50.0)	0.62
GGO	9 (45.0)	7 (35.0)	10 (50.0)	

BMI: body mass index; DP: diaphragmatic positioning; GCO: ground glass opacity; IS: incentive spirometry.

Comparison between the groups studied regarding the clinical and radiological data revealed significant improvement in G1 and G2 in all parameters at the end of follow-up. However, no significant improvement was found in G3. In G1, the mMRC dyspnoea scale improved from 1.9 ± 0.8 to 0.7 ± 0.7 (*p* < 0.001) while in G2, it changed from 1.6 ± 0.5 to 0.9 ± 0.7 (*p* < 0.001). No significant change was noted in G3. Similar findings were noted regarding 6MWT in G1 (*p* < 0.001 and G2 (*p* < 0.001), SVC in G1 (*p* < 0.001) and G2 (*p* < 0.001), FEV1/FVC in G1 (*p* < 0.001), MVV in G1 (*p* = 0.013) and G2 (*p* < 0.001), SpO2 in G1 (*p* < 0.001) and G2 (*p* < 0.001), SpO_2-_6MWT in G1 (*p* < 0.001) and G2 (*p* < 0.001), diaphragmatic thickness in G1 (*p* < 0.001) and G2 (*p* < 0.001), and diaphragmatic excursion in G1 (*p* < 0.001) and G2 *p* < 0.001). No significant changes were found in G3 regrading 6MWT, SVC, FEV1/FVC, MVV, SpO2, SpO_2-_6MWT, diaphragmatic thickness, and diaphragmatic excursion. Notably, at the end of follow-up, patients in G1 outperformed their counterparts in G2 regarding 6MWT (396.9 ± 30.8 m vs 358.0 ± 52.7), FEV1/FVC (83.8 ± 2.9 vs 81.4 ± 1.5), and diaphragmatic thickness (1.04 ± 0.24 mm vs 0.86 ± 0.1) (Table II).

**Table II:** Effect of study interventions on clinical parameters

Factor	G1 Standard care + IS *n* = 20	G2 Standard care + DB *n* = 20	G3 Standard care *n* = 20	*p*-value
mMRC, mean ± SD				
Baseline	1.9 ± 0.8	1.6 ± 0.5	1.9 ± 0.7	0.3
Follow-up	0.7 ± 0.7^*^	0.9 ± 0.7^*^	1.7 ± 0.8	< 0.001
*p*-value	< 0.001	< 0.001	0.1	–
6MWT (m), mean ± SD				
Baseline	216.2 ± 46.8	223.2 ± 41.1	202.6 ± 44.0	0.28
Follow-up	396.9 ± 30.8^*#^	358.0 ± 52.7^*^	217.9 ± 30.4	< 0.001
*p*-value	< 0.001	< 0.001	0.22	–
SVC (%), mean ± SD				
Baseline	75.1 ± 2.4	76.6 ± 2.2	76.3 ± 2.1	0.1
Follow-up	89.1 ± 3.1^*^	86.9 ± 4.2^*^	83.1 ± 3.1	< 0.001
*p*-value	< 0.001	< 0.001	0.39	–
FEV1/FVC				
Baseline	80.4 ± 1.2	80.7 ± 1.5	80.2 ± 1.9	0.65
Follow-up	83.8 ± 2.9^*#^	81.4 ± 1.5^*^	80.8 ± 2.7	0.001
*p*-value	< 0.001	0.069	0.47	–
MVV (l/m), mean ± SD				
Baseline	49.0 ± 4.5	47.0 ± 4.9	47.0 ± 4.6	0.31
Follow-up	51.1 ± 3.8^*^	49.6 ± 4.1	47.7 ± 4.1	0.037
*p*-value	0.013	< 0.001	0.41	–
SpO_2_ (%), mean ± SD				
Baseline	94.8 ± 0.8	95.1 ± 1.1	94.7 ± 0.9	0.31
Follow-up	97.9 ± 0.8^*^	97.5 ± 0.9^*^	94.9 ± 0.8	< 0.001
*p*-value	< 0.001	< 0.001	0.29	–
SpO_2-_6MWT (%), mean ± SD				
Baseline	92.0 ± 1.1^*^	92.0 ± 1.2^*^	91.9 ± 1.4	0.96
Follow-up	96.8 ± 0.7	96.6 ± 1.2	92.5 ± 0.8	< 0.001
*p*-value	< 0.001	< 0.001	0.083	–
Diaphragmatic thickness (mm), mean ± SD				
Baseline	0.52 ± 0.11	0.54 ± 0.1	0.56 ± 0.1	0.52
Follow-up	1.04 ± 0.24^*#^	0.86 ± 0.1^*^	0.6 ± 0.27	< 0.001
*p*-value	< 0.001	< 0.001	0.62	–
Diaphragmatic excursion (cm), mean ± SD				
Baseline	2.96 ± 0.18	2.98 ± 0.19	2.93 ± 0.17	0.74
Follow-up	4.53 ± 0.67^*^	4.39 ± 0.78^*^	2.98 ± 0.61	< 0.001
*p*-value	< 0.001	< 0.001	0.71	–

* Significant results versus G3;

# significant results versus G2.

6MWT: 6-minute walk test; DB: diaphragmatic breathing; FEV1: forced expiratory volume 1st second; FVC: forced vital capacity; IS: incentive spirometry; mMRC: modified Medical Research Council; MVV: maximal voluntary ventilation; SpO_2:_ oxygen saturation; SVC: slow vital capacity.

At the end of follow-up, 15 patients (75.0%) in G1, 11 patients (55.0%) in G2, and 3 patients (15.0%) in G3 showed improvement on the mMRC dyspnoea scale. Multivariate logistic regression analysis identified mild acute COVID-19 infection (OR [95% CI]: 10.79 [1.83–63.47], *p* = 0.009), use of IS (OR [95% CI]: 0.03 [0.01–0.22], *p* < 0.001), and use of DB (OR [95% CI]: 0.14 [0.03–0.76], *p* = 0.023) as significant predictors of improvement on the mMRC dyspnoea scale (Table III).

**Table III:** Predictors of improved mMRC in the patients studied

Factor	Univariate analysis			Multivariate analysis		
	OR	95%CI	*p*-value	OR	95%CI	*p*-value
Age	0.95	0.85–1.06	0.34	–	–	–
Sex	2.59	0.9–7.39	0.076	–	–	–
COVID severity	0.12	0.03–0.41	< 0.001	10.79	1.83–63.47	0.009
Radiology	0.19	0.06–0.59	0.004	0.33	0.07–1.52	0.15
Intervention						
Standard care	Reference group					
Standard care + IS	0.06	0.01–0.29	< 0.001	0.03	0.01–0.22	< 0.001
Standard care + DB	0.14	0.03–0.65	0.012	0.14	0.03–0.76	0.023

## DISCUSSION

In the present study, respiratory muscle training using IS or DB resulted in improved clinical and physical performance in patients with post-COVID-19 condition and diaphragmatic dysfunction in comparison with patients subjected to standard care alone. This improvement was particularly expressed as better mMRC dyspnoea scale, 6MWT, and oxygen saturation. These findings provide supportive data on the use of respiratory exercises in post-COVID rehabilitation for patients with shortness of breath and/or easy fatiguability. While both interventions proved to be superior to standard care, IS provided better 6MWT as compared with DB.

Conclusions of this study are in harmony with multiple reports. In the randomized study of Palau et al. (27) on patients with post COVID-19 condition, home-based inspiratory muscle training resulted in improved peak oxygen consumption and chronotropic response during exercise. Also, the study of Altmann et al. (6) documented significant improvement of pulmonary functions after inspiratory respiratory training. In another study, Villelabeitia-Jaureguizar et al. (28) concluded that low-intensity respiratory muscle training may improve respiratory muscle strength and dyspnoea. In other work, Del Corral et al. (29) found that an 8-week supervised home-based respiratory (inspiratory/expiratory) muscle training programme was effective only in improving quality of life, but not exercise tolerance in individuals with long-term post-COVID-19 symptoms. In addition, they noted that both inspiratory and respiratory muscle training programmes were effective in improving respiratory muscle function and lower limb muscle strength, but had no impact on lung function and psychological status.

In the current study, it was also found that IS muscle training showed better performance when compared with DB. In harmony with these findings, the study of Ribeiro et al. (30) noted that in patients with Parkinson’s disease and respiratory dysfunction, there was better improvement of pulmonary function after IS as compared with stacking breathing exercises. In patients with COPD, another study found that use of IS resulted in more improvement of pulmonary functions, 6MWT, and inflammatory markers as compared with deep breathing tests (31). Also, it was found that, in stroke patients, use of flow IS was associated with better pulmonary functions and maximal respiratory pressures when compared with diaphragmatic breathing and volume IS (32). Likewise, IS training after cardiac surgery was linked to better postoperative oxygen saturation and a lower rate of dyspnoea (33, 34).

The findings of the present study may be limited by the small sample size and the short duration of follow-up. However, they remain relevant and encouraging. Further confirmatory studies are strongly recommended. Moreover, patients and investigators were not blinded regarding patients’ allocation to interventional groups. To minimize the risk of bias related to this issue all assessments were performed by independent nurses or technicians who were not aware of the study purpose or patients’ allocation.

In conclusion, use of IS or DB in addition to standard care in post COVID-19 condition patients was associated with better clinical improvement as compared with standard care alone. Also, IS training resulted in better pulmonary functions and physical performance in comparison witho DB.
